# Three-Dimensional Analysis of the Characteristics of the Femoral Canal Isthmus: An Anatomical Study

**DOI:** 10.1155/2015/459612

**Published:** 2015-06-07

**Authors:** Xiu-yun Su, Jing-xin Zhao, Zhe Zhao, Li-cheng Zhang, Chen Li, Jian-tao Li, Jian-feng Zhou, Li-hai Zhang, Pei-fu Tang

**Affiliations:** ^1^Department of Orthopaedics, Chinese PLA General Hospital, No. 28, Fuxing Road, Beijing 100853, China; ^2^Department of Orthopaedics, Affiliated Hospital of the Academy of Military Medical Sciences, No. 8, Dongdajie Road, Beijing 100071, China; ^3^Department of Orthopaedics, Beijing Tsinghua Chang Gung Hospital, No. 1, Block Tiantongyuan North, Beijing 102218, China

## Abstract

*Purpose*. To establish a new approach for measuring and locating the femoral intramedullary canal isthmus in 3-dimensional (3D) space. *Methods*. Based on the computed tomography data from 204 Chinese patients, 3D models of the whole femur and the corresponding femoral isthmus tube were reconstructed using Mimics software (Materialise, Haasrode, Belgium). The anatomical parameters of the femur and the isthmus, including the femur length and radius, and the isthmus diameter and height, were measured accordingly. *Results*. The mean ratio of the isthmus height versus the femoral height was 55 ± 4.8%. The mean diameter of the isthmus was 10.49 ± 1.52 mm. The femoral length, the isthmus diameter, and the isthmus tube length were significantly larger in the male group. Significant correlations were observed between the femoral length and the isthmus diameter (*r* = 0.24, *p* < 0.01) and between the femoral length and the isthmus height (*r* = 0.6, *p* < 0.01). Stepwise linear regression analyses demonstrated that the femoral length and radius were the most important factors influencing the location and dimension of the femoral canal isthmus. *Conclusion*. The current study developed a new approach for measuring the femoral canal and for optimization of customer-specific femoral implants.

## 1. Introduction

Total hip arthroplasty (THA) and intramedullary (IM) nailing fixation have become the most successful surgical interventions among the orthopedic community [1]. Accurate preoperative morphological measurements of the femoral intramedullary canal are an essential part of the preoperative plan and facilitate the selection or design of the most suitable femoral implant [2]. As one of the most important anatomical parameters of the femur, the morphology of the femoral canal has important clinical implications when the introduction of an intramedullary device is planned. During IM nailing, the diameter of the canal must be adjusted according to the nail by reaming. When a cementless femoral stem is used during THA, it is very important to match the dimensions of the implant closely with those of the femur for primary and secondary mechanical stability [1, 3, 4].

Some narrow canals are barely able to safely accept intramedullary hardware. The intramedullary femoral guide rod can be jammed into the femoral isthmus during the process of total hip arthroplasty, and this jamming can be avoided with precise preoperative measurements regarding the diameter and location of the canal isthmus [5, 6]. Traditional measurement methods of femoral morphology are primarily based on two-dimensional (2D) images, such as anterior-posterior (AP) X-ray films [2, 7–10]. Compared to 2D measurement methods, Walsh et al. reported that the diameter of the canal was measured more accurately using coronal computed tomography (CT) images [10]. However, neither 3D geometry projected onto 2D planes nor selected CT images could accurately reflect the detailed osseous morphology of the femoral canal in 3D space; thus, the current methods are unreliable.

With the development of medical image processing and medical engineering techniques, computer-aided design (CAD) software has been used to accurately define and quantify the 3D geometry of the femoral canal with morphologic parameters. Kim et al. published the first paper measuring the femoral canal using CAD software in order to design new femoral stems that ideally distribute the stress to the bone. Since then, many related studies have been performed on this topic [11]. Using the CAD technique, Laine et al. developed a reproducible method of image processing for 3D femoral endosteal cavity shape modeling [1]. However, the intervals between the CT axial images were relatively higher than most previous studies with a range of 5–10 mm. Recently, Baharuddin et al. used thin layer CT data with 1.5 mm intervals to reconstruct 3D femoral canals [12]. Although the diameter and location of the canal isthmus were reported in their paper, the method of locating the isthmus was still based on the subjective determination of the observers.

This study established an automated and objective method of measuring the parameters and orientation of the canal isthmus in 3D space using CAD software. Ideally, this method could optimize the choice and design of femoral implants and identify difficulties that may be encountered during femoral canal-related surgeries.

## 2. Materials and Methods

This medical imaging investigation was approved by the Ethics Committee of the Chinese PLA General Hospital. Requirements for informed consent were waived due to the retrospective study design. Furthermore, the patients' data were made anonymous.

In total, 204 patients with normal femoral morphologies who underwent lower-extremity CT angiography between December 2009 and December 2012 were included in this study. Some patients were included in our previous study [25]. After evaluation by two senior orthopaedic surgeons (Li-hai Zhang and Xiu-yun Su) in an independent manner, patients with evidence of lower-extremity trauma, acetabular dysplasia, amputation, avascular necrosis of the femoral head or intervention, and femoral or pelvic implants were excluded. The demographic data, including age, gender, height, and CT data, were collected. All CT scans were performed by the Somatom sensation open CT System (Siemens AG, Erlangen, Germany) with slice thicknesses of 1.2 mm.

### 2.1. Construction of Coordinate System and 3D Model

Based on the CT data in Digital Imaging and Communication in Medicine (DICOM) format, 3D models of the whole femur and femoral canal were reconstructed using the Mimics software according to the steps described below.

**Figure 1:**
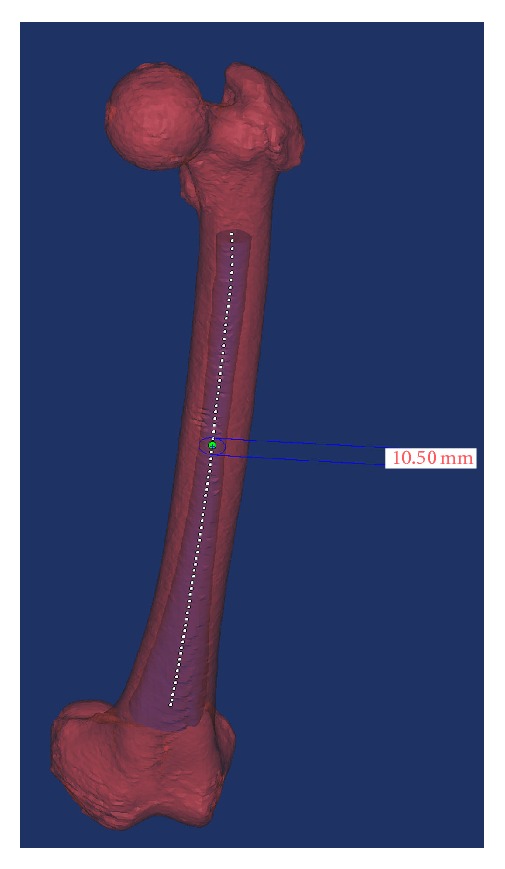
Using Mimics software, a 3D model of the femoral medullary canal was created between the lesser trochanter and the flare of the condyles. An arc centerline was fitted to the canal model using the centerline fit function. The diameters of all the inscribed circles along the arc centerlines were created with 1 mm intervals and exported to determine the isthmus location. In this case, the isthmus diameter was 10.50 mm.

After the whole femur mask was created, the cortical bone mask was created based on the threshold (ranging from 226 HU to 3071 HU) according to guidelines of the Mimics software [26]. Then, the medullary mask was created by logically subtracting the cortical mask from the whole femoral mask. The medullary canal mask was created by cutting between the lesser trochanter (LT) and the flare of the condyles (Figure 1).

Because of the inconsistent positioning of the patients' bodies during the CT scans, an idealized coordinate system was reconstructed based on the femur-specific anatomy and geometry to normalize the femur orientation.

**Figure 2:**
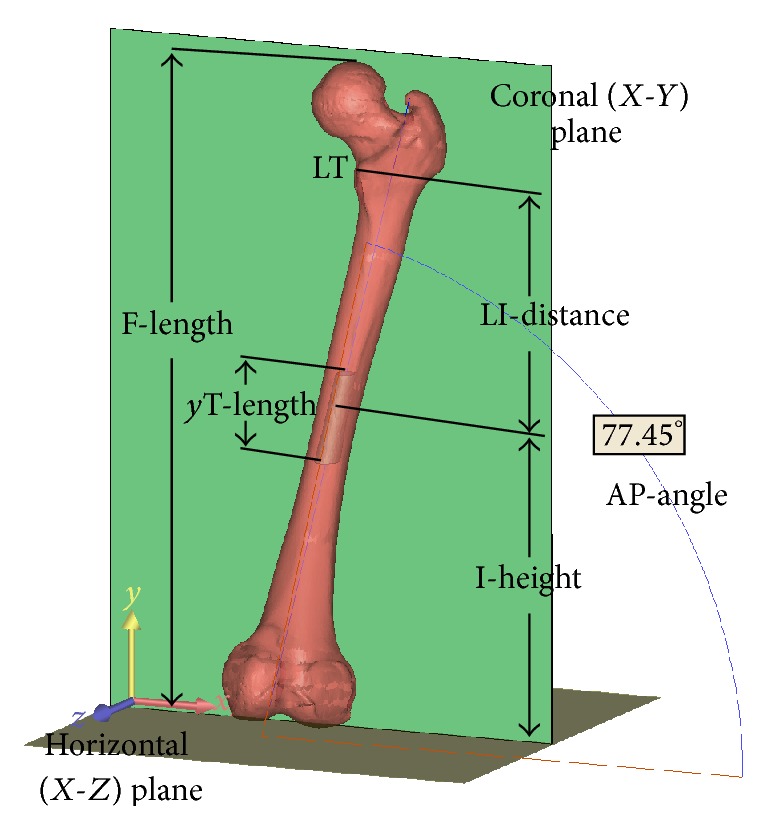
In the femur-customized coordinate system, the anatomical parameters of the femur and its isthmus tube were determined, including the femoral length (F-length), isthmus height (I-height), distance between the isthmus and LT in the *y*-axis (LI-distance), isthmus tube length in the *y*-axis (*y*T-length), isthmus tube length (T-length), and the intersecting angles between the axis of the isthmus tube and the horizontal plane in the coronary plane (AP-angle).

The 3D femur models were imported into 3DS Max software (Autodesk, San Rafael, CA, USA). Two planes representing the global Cartesian *X*-*Y* and *X*-*Z* planes were created (Figure 2). Using the MassFX tool, the virtual forces along the *z*- and *y*-axes directing to the *X*-*Y* and *X*-*Z* planes, respectively, were applied to the femur model. This application ensured that the posterior aspects of the medial and lateral femoral condyles and the greater trochanter contacted the *X*-*Y* plane (see Supplementary Material Video 1 in Supplementary Material available online at http://dx.doi.org/10.1155/2015/459612) and that the distal aspects of the medial and lateral femoral condyles contacted the *X*-*Z* plane (Supplementary Material Video 2).

The femur-customized anatomical coordinate system was constructed according to the *X*-*Y*, *X*-*Z*, and *Y*-*Z* planes, which were also defined as the coronal, horizontal, and sagittal planes, respectively. The femur length in the *y*-axis was considered to be the femoral length.

### 2.2. Parameters of the Femoral Medullary Canal Anatomy

**Figure 3:**
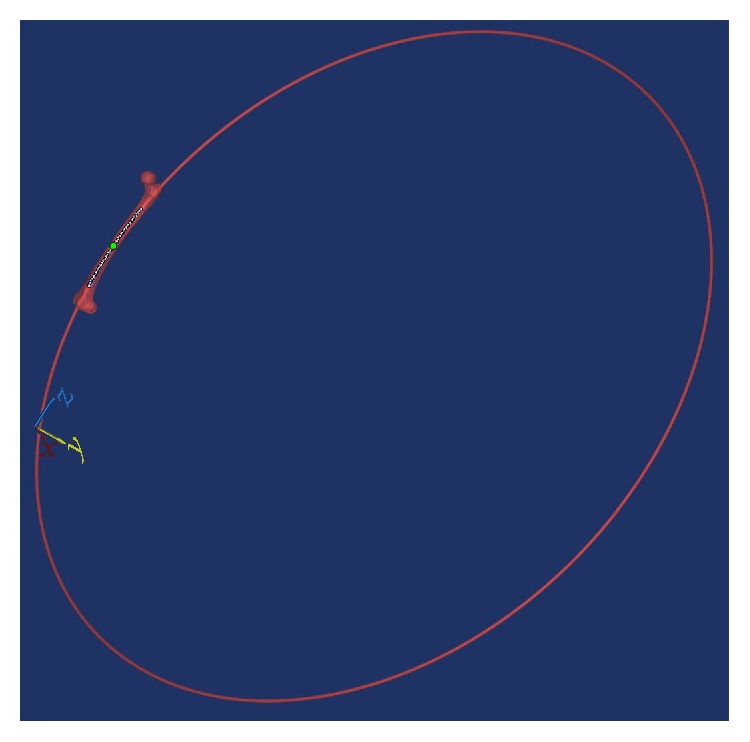
After an arc centerline was fitted to the 3D model of the femoral medullary canal, the radius of the arc was calculated and considered to represent the femur radius.

In the femur-customized coordinate system, an arc centerline was fitted to the 3D model of the femoral medullary canal using the centerline fit function of Mimics software [25]. The radius of the arc centerline was considered to be the femoral radius (Figure 3). All points on the arc centerline of the femoral medullary canal (with 1 mm intervals) were created with Mimics software (Figure 1).

The diameters of all the inscribed circles along the arc centerline were exported to identify the three smallest continuous inscribed circles. Among these three circles, the smallest diameter was considered to be the isthmus diameter, and the corresponding center was considered to be the femoral canal isthmus. The distance between the femoral canal isthmus and the center of the LT in the *y*-axis was recorded. To locate the position of the LT in 3D space, we used the method according to Masjedi et al. [27] in which the LT is selected and fitted to a sphere using the sphere fitting tool of the 3-matic. The center of the sphere was considered to be the center of the LT.

The 3D model of the femoral medullary canal isthmus was constructed and created after including all the previously mentioned inscribed circles close to the canal isthmus with the diameters within 1 mm plus the isthmus diameter, which was termed the isthmus tube. The centers of the inscribed circles at the two ends of the isthmus tube were termed points P1 and P2, respectively. The midpoint between points P1 and P2 was determined and considered to be the midpoint of the isthmus tube (Figure 2).

**Figure 4:**
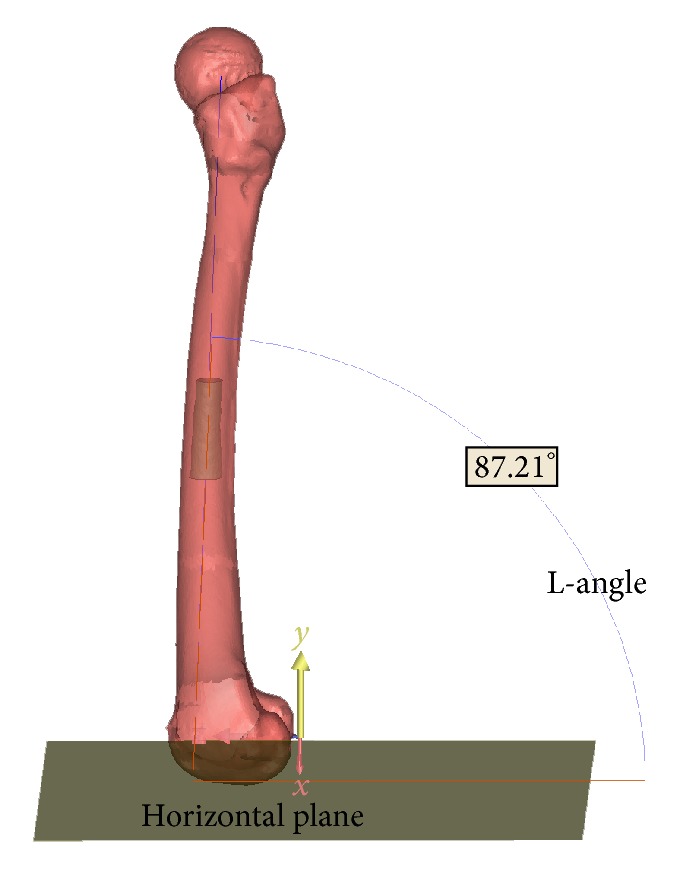
In the femur-customized coordinate system, the intersection of the angles between the axis of the isthmus tube and the horizontal plane in the sagittal plane was termed the lateral angle of the isthmus tube (L-angle).

The locations of the canal isthmus and isthmus tube in the femur were expressed as the distances from the center of the isthmus to the horizontal plane and the distance from the midpoint of the isthmus tube to the horizontal plane, respectively. The length of the isthmus tube was considered to be the distance between points P1 and P2. The length of the isthmus tube in the *y*-axis was considered to be the distance between points P1 and P2 in the *y*-axis. The line connecting P1 and P2 was considered to be the axis of the isthmus tube. The direction of the isthmus tube in 3D space was expressed as the intersection of the angles between the axis of the isthmus tube and the horizontal plane in the coronary and sagittal planes. These angles were termed the anterior-posterior- (AP-) angle and the lateral angle of the isthmus tube, respectively (Figures 3 and 4).

### 2.3. Reliability Study and Anatomical Parameters Calculation

In this study, only the selection of the medullary canal between the LT and flare of the condyles and the location of the center of the LT were manipulated manually. These results could influence the radius of the femur and the distance between the LT and isthmus. Thus, a reliability study was performed to evaluate the intra- and interobserver reliability of this method for calculating the femur radius and distance between the LT and isthmus.

Based on Walter et al.'s method [28] to determine the required sample size based on the number of replicates and the estimated expected intraclass correlation coefficient (ICC), when the minimal acceptable and expected ICC were 0.80 and 0.95, respectively, a sample size greater than 12 for a test-retest design (*k* = 2) was estimated (*α* = 0.05 and *β* = 0.20). Thus, 20 femurs were selected and were enough in this reliability study. One independent orthopaedic doctor (Xiu-yun Su) repeated the measurements of the radiuses of arbitrary 20 femoral canals two times in a randomized order with a minimum of 24 h interval. Another two doctors (Jian-feng Zhou and Jian-tao Li) measured the same samples in a randomized order and an independent manner. Two-way random and two-way fixed model of Shrout and Fleiss were selected to assess the inter- and intraobserver reliability.

After validity of the established measurement method was confirmed, the anatomical parameters of the femurs were extracted by Xiu-yun Su and Zhe Zhao, and the anatomical parameters of the canals were measured by Chen Li and Jian-feng Zhou. The abbreviations of the anatomical parameters were listed in Table 1.

### 2.4. Statistical Analysis

After verifying the normal distribution of the data by the Kolmogorov-Smimov test, the data with or without normal distribution were tested using Student's* t*- or Mann-Whitney *U* tests, respectively. Independent and paired samples Student's* t*-test compared between-gender or between-laterality differences, respectively. Correlations between variables were determined using Pearson's correlation analysis. After verification of the absence of the colinearity among the independent variables, stepwise linear regression model was applied to investigate the influential factors of the anatomical parameters of the isthmus. Statistical significance was set at *p* < 0.05. Statistical analyses were performed using SPSS 20 (SPSS Inc., Chicago, IL, USA).

## 3. Results

The population characteristics and the anatomical parameters of the femur and isthmus are summarized in Table 2. The ratio of the isthmus height relative to the femoral height was 55 ± 4.8%. The diameter of the isthmus was 10.49 ± 1.52 mm.

Table 3 demonstrates that both the inter- and intraobserver reliability of the measurement methods for calculating the femoral radius and locating the LT were very high. Independent samples *t*-tests revealed significant differences in age, height, weight, F-radius, F-length, I-diameter, *y*T-length, I-height, and L-angle between genders (Table 2). A paired samples *t*-test indicated that the F-length (*p* = 0.015), F-radius (*p* < 0.01), I-diameter (*p* < 0.01), and L-angle (*p* = 0.011) on the left side were significantly larger than the right side (Table 4).

Pearson's correlation analyses were performed to detect correlations among the general characteristics of the included population and the anatomical parameters of the femur and canal isthmus (Table 5). The F-length and I-diameter (*r* = 0.24, *p* < 0.01) and the F-length and I-height (*r* = 0.6, *p* < 0.01) were significantly correlated.

Stepwise linear regression analyses were applied with the I-diameter, *y*T-length, LI-distance, and I-height as dependent variables. The characteristics or parameters that were significantly correlated with each dependent variable were set as the independent variables. Using the final regression model, the I-diameter was calculated as 6.542 + 0.015 × the F-length + 0.01 × the *y*T-length − 0.036 × AP-angle (*R* ^2^ = 0.087). Similarly, the I-height was calculated as 61.741 − 0.92 × the LI-distance + 0.801 × the F-length − 0.729 × the L-angle (*R* ^2^ = 0.960). The LI-distance was calculated as 8.499 − 0.985 × the I-height + 0.795 × the F-length (*R* ^2^ = 0.937). The *y*T-length was calculated as 72.683 + 0.429 × the T-length − 0.014 × the F-radius + 1.443 × the I-diameter − 25.124 × the height (*R* ^2^ = 0.453).

## 4. Discussion

Previous studies regarding the morphology of the femur and its canal were performed primarily by anthropologists and anatomists [29–32]. To determine the endosteal dimensions of the femur, the most common method previously used was to cut the femur into several cross sections at certain intervals and measure the diameter of the medullary canal with a caliper. Wei et al. and Dokládal studied cross sections of the human femoral intramedullary canal and divided the morphological variations into different types. The authors demonstrated high variability and the absence of uniformity of the canal shape along the femoral diaphysis [30, 31]. Then, the morphology of the femoral canal was studied primarily by orthopedic surgeons and biomedical engineers for preoperative planning of IM nailing, arthroplasty surgery, or designing more suitable femoral implants that fit precisely to the 3D nature of the intramedullary canal.

The radiograph is the most common tool for determining the dimensions and location of the femoral canal [2, 7, 9, 13, 14, 20], which are usually expressed as the diameter of the isthmus and its position relative to the LT, respectively. The measurement results are highly variable among authors.  Table 6 summarizes the measurement methods and results regarding the femoral canal isthmus according to previous studies.

Because AP or lateral radiographs can only provide a rough 2D approximation of the actual dimensions of the femoral canal, the limitations of traditional radiology are obvious regarding measurements of the morphologic parameters of the irregular femoral canal compared to 3D space. Considering the remarkable variability among individuals regarding endosteal morphology, a standardized measurement method based on 2D radiographs is not possible. The measurement methods based on the CT data might provide more precise descriptions [4, 12, 13, 16, 18, 19, 22]. To design a better intramedullary femoral implant, Kim et al. used CAD software to calculate the dimensions of the femoral intramedullary canal in order to improve the implant-canal contact [11, 33]. Laine et al. developed a CT-based image processing computer program to determine the 3D femoral endosteal cavity shape [1]. After CT scanning 50 femurs at 10 mm intervals, the average isthmus diameter in the AP direction was 11.06 mm, and the average distance between the isthmus and LT was 110 mm [16]. Khang et al. analyzed 3D femur models from 38 cadavers and 200 volunteers based on CT scans at 4 mm intervals and revealed that the average isthmus width was 12.2 mm, and the average distance between the isthmus and LT was 115.5 mm [4].

Using a technique that was very similar to ours, Baharuddin et al. reported that the isthmus width and the distance between the isthmus and LT were 9.73 and 112.83 mm, respectively [12]. However, their measurements were performed along the femur at 10 mm intervals. Except for the study by Baharuddin et al., the CT measurements in the previous studies were all performed at 5–10 mm intervals, which might lead to inaccurate descriptions of the isthmus dimensions and location. Additionally, even with the CAD techniques and 3D CT data, the position of the isthmus in the previous studies was expressed as its position relative to the LT. Thus, the actual position and orientation of the isthmus in 3D space were still unknown. Furthermore, regarding the irregular femoral canal shape, most previous researchers measured the mediolateral (ML) or AP width of the isthmus based on either AP or lateral radiographs or CT images, which are still 2D measurements. These authors concluded that the isthmus AP width was greater than the ML width, such as in Wang et al. [21]. However, the tips of most femoral implants were designed with hemisphere shapes. Thus, the inscribing circle of the canal along the canal's centerline might be a more important and reliable reference for implant designs.

To determine the position and dimensions of the isthmus more precisely, CT scans at 1.2 mm intervals were obtained. We reconstructed 3D femur and canal models. We used Mimics software to fit the centerline of the 3D canal model into an arc. The smallest inscribing circles along the arc centerline of the canal were calculated at 1 mm intervals. Among these circles, the smallest one was considered to be the isthmus. Furthermore, to describe the anatomy of the femoral isthmus in 3D space, an isthmus tube was reconstructed, and the direction of the tube was expressed as the intersecting angles between its axis and the horizontal plane in the coronary and sagittal planes, respectively. These results indicate that it might be inappropriate to measure and determine the orientation of the isthmus or the canal from AP or lateral radiographs.

Based on previous studies, the remarkable variability of isthmus parameters is consistent with the observation that the geometry of the isthmus is determined by a large number of factors, including the general characteristics of the individual and femoral anatomical parameters. These factors create unique isthmus geometries, like the other characteristics of femoral anatomy. Although a paired sample *t*-test indicated that the femoral length, radius, and isthmus diameter of the left side were significantly larger than the right side, the average between-laterality differences were 0.63 mm, 57.98 degrees, and 0.2 mm, respectively. These differences may not clinically impact implant design and preoperative assessments. However, these differences require further evaluations in future studies.

Onoue et al. suggested that older patients have larger isthmus diameters [13]. Milligan et al. reported that this correlation only exists among females (*r* = 0.31, *p* < 0.001) [23]. This observation agrees with Noble et al. [7]. However, we observed no correlations between age and isthmus diameter in males, females, or the total population. We also tried to establish linear relationships between the isthmus parameters and general characteristics or other femoral anatomical parameters. The stepwise linear regression analyses demonstrated that the femoral length and radius and the AP and lateral angles of the isthmus tube were the most important factors influencing the dimensions and location of the femoral canal isthmus. The isthmus diameter is increased when the femoral length is increased or the AP-angle of the isthmus tube is decreased. When the L-angle of the isthmus tube is decreased, the isthmus position is much higher. The length of the isthmus tube in the *y*-axis was influenced by the length of the isthmus tube in 3D space and the femoral radius and body height. When the F-radius or height increased, the *y*T-length decreased. In addition, a correlation analysis demonstrated that the anatomical parameters of the isthmus were influenced by each other. The lateral angle of the isthmus tube was negatively correlated with the height of the isthmus and positively correlated with the distance between the isthmus and LT in the *y*-axis. When the isthmus diameter increased, the isthmus tube in the *y*-axis also increased.

This study had some limitations. The included population primarily consisted of elderly Chinese individuals. These measurements may not be representative of other ages or populations in other countries. In addition, there might be other important influential factors of the femoral canal isthmus, from either the general characteristics or the femoral anatomy, rather than the factors included in the present study. Further studies are needed to be performed to find out more meaningful factors to evaluate the anatomy of the canal isthmus.

## 5. Conclusion

In conclusion, the current study developed a new measurement approach to evaluate the anatomical morphology of the femoral canal isthmus and determined the most influential factors on the location and dimension of the isthmus. Furthermore, this study was useful for establishing the methodological basis for preoperative assessments of the femur canal-related surgeries and developments of the femoral implants more suitable for the anatomy of old Chinese.

## Supplementary Material

Description of video 1: This was a y-axial view. After the virtual force along the z axis directing to the X-Y plane was applied to the femur model, the femur was placed on the coronal (X-Y) plane. This application could ensure that the posterior aspects of the medial and lateral femoral condyles and the greater trochanter contacted the X-Y plane.
Description of video 2: This was a z-axial view. After the previous step in video 1, the virtual force along the Y axis directing to the X-Z plane was applied to the femur model. This application could ensure that the distal aspects of the medial and lateral femoral condyles contacted the X-Z plane.

## Figures and Tables

**Table 1:** The abbreviations of the anatomical parameters of the femurs and canals.

Items	Abbreviations
Femoral length	F-length
Femoral radius	F-radius
Isthmus diameter	I-diameter
Height of the isthmus	I-height
Ratio of the height of the isthmus versus the femoral length	I-index
Distance between the isthmus and the LT in the *y*-axis	LI-distance
Length of the isthmus tube in the *y*-axis	*y*T-length
Ratio of the height of the midpoint of the isthmus tube versus the femoral length	T-index
Length of the isthmus tube	T-length
AP-angle of the isthmus tube	AP-angle
Lateral angle of the isthmus tube	L-angle

**Table 2:** The main characteristics of the population and the anatomical parameters of the femur and the isthmus.

Items	Male (*n* = 282)		Female (*n* = 126)		Student's *t*-test		Total (*n* = 408)	
	Mean ± SD	Range	Mean ± SD	Range	*t*	*p*	Mean ± SD	Range
Age (y)	64.84 ± 12.95	15–85	69.67 ± 8.5	50–85	—	0.002^*∗*^	66.33 ± 12	15–85
Height (m)	1.69 ± 0.059	1.48–1.84	1.59 ± 0.061	1.40–1.77	16.929	<0.001	1.66 ± 0.078	1.40–1.84
Weight (kg)	68.59 ± 9.9	43–97	62.35 ± 9.9	37–86	5.884	<0.001	66.66 ± 10.3	37–97
F-length (mm)	437.75 ± 19.81	386–485	406.70 ± 22.04	352–487	14.121	<0.001	428.16 ± 25.03	352–487
F-radius (mm)	1007.30 ± 205.7	616–2029	880.52 ± 171.32	511–1375	6.044	<0.001	968.15 ± 204.12	511–2029
I-diameter (mm)	10.68 ± 1.39	6.60–16.20	10.05 ± 1.71	6.00–14.00	3.93	<0.001	10.49 ± 1.52	6.00–16.20
I-height (mm)	242.6 ± 23	166.22–317.6	221.66 ± 25.46	137.15–284.8	8.21	<0.001	236.13 ± 25.66	137.15–317.6
I-index	0.55 ± 0.046	0.37–0.75	0.54 ± 0.052	0.35–0.68	1.95	0.052	0.55 ± 0.048	0.35–0.75
LI-distance (mm)	117.06 ± 21.69	34.16–197.47	114.66 ± 20.87	63.98–186.25	1.04	0.3	116.31 ± 21.45	34.16–197.47
*y*T-length (mm)	57.63 ± 22.2	7.21–136.83	61.29 ± 22.33	10.71–122.19	−1.54	0.12	58.76 ± 22.28	7.21–136.83
T-index	0.55 ± 0.04	0.42–0.69	0.55 ± 0.044	0.37–0.66	0.765	0.45	0.55 ± 0.04	0.37–0.69
T-length (mm)	60.87 ± 37.11	7.39–545.45	62.44 ± 22.7	11.28–123.97	−0.440	0.66	61.36 ± 33.32	7.39–545.45
AP-angle (degrees)	80.57 ± 4.55	16.27–89.23	81.21 ± 2.79	71.39–89.17	−1.459	0.15	80.77 ± 4.1	16.27–89.23
L-angle (degrees)	84.69 ± 1.96	69.16–90.33	83.89 ± 1.39	79.94–89.38	4.183	<0.001	84.44 ± 1.84	69.16–90.33

^*∗*^The data of the age was nonnormal distribution and was tested using the Mann-Whitney *U* test.

**Table 3:** Reliability study results.

Items	Intraobserver		Interobserver	
	ICC	95% CI	ICC	95% CI
F-radius	0.998	0.995–0.999	0.997	0.994–0.998
LI-distance	0.995	0.993–0.997	0.994	0.991–0.996

**Table 4:** Paired samples *t*-test of the anatomical parameters of the femurs and the isthmuses between different lateralities (*n* = 204, mean ± SD).

Items	Left side (mean ± SD)	Right side (mean ± SD)	Difference (mean ± SD)	*t*	*p*
F-length (mm)	428.48 ± 24.92	427.84 ± 25.18	0.63 ± 3.69	2.446	0.015
F-radius (mm)	997.14 ± 217.56	939.16 ± 185.78	57.98 ± 95.79	8.645	<0.001
I-diameter (mm)	10.59 ± 1.51	10.39 ± 1.53	0.2 ± 0.61	4.697	<0.001
I-height (mm)	236.95 ± 26.43	235.31 ± 24.89	1.64 ± 21.59	1.085	0.279
I-index	0.55 ± 0.051	0.55 ± 0.045	0.0033 ± 0.051	0.917	0.36
LI-distance (mm)	115.15 ± 22.26	117.48 ± 20.59	−2.33 ± 21.66	−1.539	0.125
*y*T-length (mm)	58.51 ± 22.24	59.01 ± 22.38	−0.49 ± 23.66	−0.298	0.766
T-index	0.55 ± 0.04	0.55 ± 0.044	0.0037 ± 0.031	1.692	0.092
T-length (mm)	62.06 ± 40.8	60.65 ± 23.66	1.41 ± 42.42	0.475	0.635
AP-angle (degrees)	80.99 ± 2.83	80.54 ± 5.05	0.46 ± 5.2	1.254	0.211
L-angle (degrees)	84.58 ± 2	84.31 ± 1.65	0.28 ± 1.54	2.565	0.011

**Table 5:** The correlation analysis between the main characteristics of the population and the anatomical parameters of the femur and the isthmus.

Pearson *r*	Age	Height	Weight	F-length	F-radius	I-diameter	I-height	LI-distance	*y*T-length	T-length	AP-angle	L-angle	I-index	T-index
Age	1	−0.345^*∗∗*^	−0.371^*∗∗*^	−0.290^*∗∗*^	−0.072	−0.006	−0.272^*∗∗*^	0.059	0.05	0.042	0.059	−0.079	−0.141^*∗∗*^	−0.078
Height	*p *< 0.001	1	0.537^*∗∗*^	0.841^*∗∗*^	0.327^*∗∗*^	0.220^*∗∗*^	0.512^*∗∗*^	0.176^*∗∗*^	−0.132^*∗∗*^	−0.038	−0.110^*∗*^	0.175^*∗∗*^	0.074	0.052
Weight	*p *< 0.001	*p* < 0.001	1	0.423^*∗∗*^	0.110^*∗*^	0.112^*∗*^	0.323^*∗∗*^	0.007	0.019	0.011	−0.074	0.061	0.120^*∗*^	0.054
F-length	*p *< 0.001	*p* < 0.001	*p* < 0.001	1	0.357^*∗∗*^	0.240^*∗∗*^	0.600^*∗∗*^	0.221^*∗∗*^	−0.059	0.037	−0.102^*∗*^	0.221^*∗∗*^	0.083	0.025
F-radius	*p* = 0.145	*p* < 0.001	*p* = 0.026	*p* < 0.001	1	−0.016	0.239^*∗∗*^	0.05	−0.122^*∗*^	0.059	−0.036	0.309^*∗∗*^	0.06	0.054
I-diameter	*p* = 0.907	*p* < 0.001	*p* = 0.024	*p* < 0.001	*p* = 0.751	1	0.071	0.118^*∗*^	0.129^*∗∗*^	0.074	−0.109^*∗*^	0.113^*∗*^	−0.07	−0.118^*∗*^
I-height	*p *< 0.001	*p* < 0.001	*p* < 0.001	*p* < 0.001	*p* < 0.001	*p* = 0.154	1	−0.622^*∗∗*^	0.076	0.093	0.001	−0.246^*∗∗*^	0.844^*∗∗*^	0.619^*∗∗*^
LI-distance	*p* = 0.238	*p* < 0.001	*p* = 0.892	*p* < 0.001	*p* = 0.317	*p* = 0.017	*p* < 0.001	1	−0.122^*∗*^	−0.074	−0.068	0.477^*∗∗*^	−0.916^*∗∗*^	−0.679^*∗∗*^
*y*T-length	*p* = 0.315	*p* = 0.007	*p* = 0.702	*p* = 0.237	*p* = 0.014	*p* = 0.009	*p* = 0.127	*p* = 0.014	1	0.644^*∗∗*^	0.078	−0.05	0.148^*∗∗*^	0.189^*∗∗*^
T-length	*p* = 0.402	*p* = 0.446	*p* = 0.832	*p* = 0.457	*p* = 0.237	*p* = 0.135	*p* = 0.059	*p* = 0.134	*p* < 0.001	1	−0.004	0.06	0.100^*∗*^	0.155^*∗∗*^
AP-angle	*p* = 0.233	*p* = 0.026	*p* = 0.135	*p* = 0.04	*p* = 0.464	*p* = 0.028	*p* = 0.984	*p* = 0.173	*p* = 0.115	*p* = 0.93	1	0.104^*∗*^	0.067	0.101^*∗*^
L-angle	*p* = 0.112	*p* < 0.001	*p* = 0.222	*p* < 0.001	*p* < 0.001	*p* = 0.023	*p* < 0.001	*p* < 0.001	*p* = 0.317	*p* = 0.224	*p* = 0.035	1	−0.443^*∗∗*^	−0.588^*∗∗*^
I-index	*p* = 0.004	*p* = 0.133	*p* = 0.016	*p* = 0.096	*p* = 0.23	*p* = 0.16	*p* < 0.001	*p* < 0.001	*p* = 0.003	*p* = 0.044	*p* = 0.179	*p* < 0.001	1	0.760^*∗∗*^
T-index	*p* = 0.114	*p* = 0.299	*p* = 0.273	*p* = 0.621	*p* = 0.277	*p* = 0.018	*p* < 0.001	*p* < 0.001	*p* < 0.001	*p* = 0.002	*p* = 0.042	*p* < 0.001	*p* < 0.001	1

^*∗∗*^Indicating the *p* value <0.01; ^*∗*^indicating the *p* value <0.05.

**Table 6:** Summary of the measurement methods and results of the femoral canal isthmus reported in previous studies.

Author	Year	Number	Subject	Origin	I-diameter (mean ± SD, mm)	LI-distance (mean ± SD, mm)	Methods
Onoue et al. [13]	1979	160	Patients	Japanese	10.9 ± 1.9	NA	AP radiograph
					11.2 ± 2.3 (ML); 14.2 ± 2.9 (AP)	NA	CT scan
Noble et al. [2]	1988	200	Cadavers	Caucasian	12.3 ± 2.3 (ML); 16.9 ± 3.5 (AP)	113.4 ± 16.4	Radiograph
Rubin et al. [3]	1992	32	Cadavers	Swiss	13.1 ± 2.1	105.7 ± 17.9	AP radiograph
Noble et al. [7]	1995	80	Cadavers	Caucasian	12.1 ± 2 (ML); 15.7 ± 2.9 (AP)	119.4 ± 16.2	Radiograph
Husmann et al. [14]	1997	310	Patients	Caucasian	11.6 ± 2.7	NA	AP radiograph
Bo et al. [15]	1997	100	Patients	Japanese	11.90 ± 2.60 (ML); 10.40 ± 2.60 (AP)	73.00 ± 18.90	3D reconstruction
Laine et al. [16]	2000	50	Cadavers	Finnish	11.06 ± 1.88 (ML); 14.09 ± 2.81 (AP)	110 ± 15	3D reconstruction
Massin et al. [17]	2000	200	Patients	French	12.40 ± 2.30	NA	AP radiograph
Mahaisavariya et al. [18]	2002	108	Cadavers	Thai	10.05 ± 1.81	112.93 ± 17.96	3D reconstruction
Khang et al. [4]	2003	200	Volunteers	Korean	11.0 ± 2.0 (ML); 12.6 ± 2.3 (AP)	117.0 ± 4.5	3D reconstruction
Noble et al. [19]	2003	53	Volunteers	Caucasian	12.7 ± 2.4 (ML); 13.9 ± 3.2 (AP)	109.3 ± 15.8	3D reconstruction
Atilla et al. [20]	2007	114	Cadavers	Turkish	10.7 ± 1.84	104.0 ± 27.9	AP radiograph
Wang et al. [21]	2009	18	Cadavers	Chinese	12.02 ± 1.13 (ML); 14.67 ± 1.52 (AP)	NA	Canal cast mold
Uzel et al. [9]	2011	106	Patients	Mixed	13.6 ± 0.90 (Afro-Caribbean); 15.3 ± 1.96 (French)	NA	AP radiograph
Rawal et al. [22]	2012	98	Patients	Indian	9.02 ± 1.92 (ML); 11.47 ± 2.11 (AP)	107.8 ± 9.73	3D reconstruction
Milligan et al. [23]	2013	1685	Patients	Caucasian	12.69 ± 2.44 (F); 13.27 ± 2.21 (M)	NA	AP radiograph
Baharuddin et al. [12, 24]	2014	60	Patients	Malay	9.73 ± 1.80 (ML); 13.12 ± 2.46 (AP)	112.83 ± 11.80	3D reconstruction
Our study	2015	408	Patients	Chinese	10.49 ± 1.52	116.31 ± 21.45	3D reconstruction

ML: mediolateral width; AP: anteroposterior width; F: female; M: male.
